# Markers of infection, breast-feeding and childhood acute lymphoblastic leukaemia

**DOI:** 10.1054/bjoc.2000.1495

**Published:** 2000-11-22

**Authors:** C Infante-Rivard, I Fortier, E Olson

**Affiliations:** Department of Epidemiology and Biostatistics and Occupational Health, Faculty of Medicine, McGill University, 1130 Pine Avenue West, Montréal, Province of Québec, H3A 1A3, Canada; Research Centre, Centre Hospitalier Universitaire Mère-Enfant, Hôpital Sainte-Justine, Université deontréal, 3175 Chemin Côte Ste-Catherine, Montréal, Province of Québec, H3T 1C5, Canada; Departement of Preventive and Social Medicine, Faculty of Medicine, Université de Montréal, Montréal, Canada

**Keywords:** childhood leukaemia, infection, breast-feeding, day-care attendance, population mixing

## Abstract

Infections are suspected to play a role in the aetiology of childhood leukaemia. In 1989–95, we evaluated the relation between childhood acute lymphoblastic leukaemia and pre- and postnatal markers of exposure to infection, as well as breast-feeding. A population-based case-control study was carried out in certain regions of Québec, Canada, in 1989–95 including 491 incident cases diagnosed between 1980 and 1993 and aged between 0 and 9 years. An identical number of healthy controls matched for age, sex and region of residence at the date of diagnosis was included. Having older siblings, mother's use of antibiotics during pregnancy, and being born second or later were all associated with increased risk of leukaemia while early day-care attendance (odds ratio (OR) = 0.49; 95% CI 0.31–0.77), and breast-feeding (OR = 0.68; 95% CI 0.49–0.95) were significantly protective. A marker of population mixing was not a risk factor. When including all variables defining family structure in a model, having older siblings at time of diagnosis was a risk factor among children diagnosed before 4 years of age (OR = 4.54; 95% CI 2.27–9.07) whereas having older siblings in the first year of life was protective among children diagnosed at 4 years of age or later (OR = 0.46; 95% CI 0.22–0.97). © 2000 Cancer Research Campaign http://www.bjcancer.com


*British Journal of Cancer* (2000) **83**(11), 1559–1564
© 2000 Cancer Research Campaign
doi: 10.1054/ bjoc.2000.1495, available online at http://www.idealibrary.com on IDEAL®

http://www.bjcancer.com

### Markers of infection, breast-feeding and childhood acute lymphoblastic leukaemia

**C Infante-Rivard[1,2], I Fortier[2,3] and E Olson[2]**

[1]Department of Epidemiology and Biostatistics and Occupational Health, Faculty of Medicine, McGill University, 1130 Pine Avenue West, Montréal, Province of Québec, Canada, H3A 1A3; [2]Research Centre, Centre Hospitalier Universitaire Mère-Enfant, Hôpital Sainte-Justine, Université de Montréal, 3175 Chemin Côte Ste-Catherine, Montréal, Province of Québec, Canada, H3T 1C5; [3]Departement of Preventive and Social Medicine, Faculty of Medicine, Université de Montréal, Montréal, Canada

Summary Infections are suspected to play a role in the aetiology of childhood leukaemia. In 1989–95, we evaluated the relation between childhood acute lymphoblastic leukaemia and pre- and postnatal markers of exposure to infection, as well as breast-feeding. A population-based case-control study was carried out in certain regions of Québec, Canada, in 1989–95 including 491 incident cases diagnosed between 1980 and 1993 and aged between 0 and 9 years. An identical number of healthy controls matched for age, sex and region of residence at the date of diagnosis was included. Having older siblings, mother's use of antibiotics during pregnancy, and being born second or later were all associated with increased risk of leukaemia while early day-care attendance (odds ratio (OR) = 0.49; 95% CI 0.31–0.77), and breast-feeding (OR = 0.68; 95% CI 0.49–0.95) were significantly protective. A marker of population mixing was not a risk factor. When including all variables defining family structure in a model, having older siblings at time of diagnosis was a risk factor among children diagnosed before 4 years of age (OR = 4.54; 95% CI 2.27–9.07) whereas having older siblings in the first year of life was protective among children diagnosed at 4 years of age or later (OR = 0.46; 95% CI 0.22–0.97). © 2000 Cancer Research Campaign http://www.bjcancer.com

**Keywords:** childhood leukaemia; infection; breast-feeding; day-care attendance; population mixing

Infections are suspected to play a role in the aetiology of childhood leukemia. Greaves (1997) has suggested that the pattern and timing of non-specific infections may be important; early stimulation of the immune system would promote adequate modulation, increasing the appropriateness of the response to later infections. In some possibly susceptible individuals, an inappropriate response of the immune system could increase the proliferation of premalignant clones and enhance the risk of leukaemia.

Different markers of childhood exposure to infections have been investigated. Population mixing of different types, indicative of increased levels of contact between individuals, has been associated with childhood leukaemia (Alexander et al, 1990; Kinlen, 1995; Kinlen et al, 1995; Petridou et al, 1996; Stiller and Boyle, 1996; Alexander et al, 1997; Kinlen, 1997; Dickinson and Parker, 1999). Improved hygiene conditions (as measured by a decreased prevalence of hepatitis A viral infection in the population) have been associated with a higher rate of childhood leukaemia (Smith et al, 1998). Presence of neonatal infections (McKinney et al, 1999) and number of ear infections during infancy (Neglia et al, 2000) have been associated with a reduced risk of acute lymphoblastic leukaemia (ALL). A protective effect on ALL of *Haemophilus influenzae* b vaccination (conjugate vaccine) but not of other types of vaccines was observed (Groves et al, 1999). Day-care attendance was found to be inversely associated with the risk of childhood leukaemia in one study (Petridou et al, 1993) while two other studies reported no association (Groves et al, 1999; Neglia et al, 2000). Higher risk of ALL for first-born children (Ross et al, 1994) was reported while no significant associations were observed in other studies (MacMahon, 1992; Westergaard et al, 1997; Neglia et al, 2000). Finally, an association between childhood leukaemia and viral infection during pregnancy was observed in one study (Roman et al, 1997) but not in another (McKinney et al, 1999), neither finding an association with antibiotic use during pregnancy.

The present study assessed the relation between childhood acute lymphoblastic leukaemia and pre- and postnatal markers of exposure to infection, as well as breast-feeding.

*Received 16 February 2000*
*Revised 21 July 2000*
*Accepted 11 August 2000*

*Correspondence to:* C Infante-Rivard

### MATERIAL AND METHODS

### Case ascertainment

During the study period 1989–95, we aimed to cover all cases of ALL diagnosed below age 10 between 1980 and 1993 in certain regions of the Province of Québec. Cases were recruited from tertiary care centres, equivalent to population-based ascertainment. Because cancer care is covered under the universal health plan, we believe that a negligible number of children, if any, were treated outside the province. The regions included were large, the smallest 8500 km$^2$ and the largest over 104 000 km$^2$. To reduce costs, we excluded the 5 less populated and most distant regions, which included about 10% of the provincial population. For similar reasons, from 1991 to 1993, only cases from the Metropolitan Montréal region (comprising 3 regions and about 60% of the provincial population) were included.

**Table 1** Characteristics of cases with acute lymphoblastic leukaemia and their controls[a]

| Factor | Controls (*n* and %) | Cases (*n* and %) |
|---|---|---|
| Mother's level of schooling | | |
| College or University | 188 (38.3) | 168 (34.2) |
| Secondary school | 279 (56.9) | 291 (59.2) |
| None, primary | 23 (4.7) | 32 (6.5) |
| Family income at diagnosis[b] | | |
| ≥ 40 000 | 147 (31.2) | 150 (31.0) |
| 10 000–39 000 | 297 (63.2) | 307 (63.5) |
| < 10 000 | 26 (5.5) | 26 (5.3) |
| Maternal age[c] | | |
| ≤ 35 | 470 (95.7) | 461 (93.9) |
| > 35 | 21 (4.2) | 30 (6.1) |
| Paternal age[c] | | |
| ≤ 40 | 467 (95.5) | 472 (96.3) |
| < 40 | 22 (4.5) | 18 (3.6) |
| Race of mother | | |
| White | 474 (96.5) | 466 (94.9) |
| Black | 11 (2.2) | 5 (1.0) |
| Other | 6 (1.2) | 20 (4.0) |
| Maternal smoking[d] | | |
| None | 316 (64.3) | 303 (61.7) |
| 1–20 cigarettes daily | 123 (25.0) | 136 (27.7) |
| > 20 cigarettes daily | 52 (10.6) | 52 (10.6) |

[a]Maximum number of cases and controls was 491, each. [b]In Canadian dollars (information available for 470 controls and 483 cases). [c]At birth of index child. [d]In the first trimester of the pregnancy.

ALL (International Classification of Diseases. Ninth Revision (ICD-9) coding 204.0) was diagnosed on the basis of standard clinical and biological criteria by an oncologist or a haematologist. To identify cases, we used: (1) hospitalization data from the provincial government's computerized discharge data files; (2) hospitalization censuses from the respective hospitals (we checked all medical records with a relevant discharge diagnosis); (3) lists maintained by haematology-oncology laboratories of histological data for cases; and (4) at the largest paediatric centre in the province (Hôpital Sainte-Justine, Montréal), medical records, distinct from the hospital medical record, which are maintained in the oncology outpatient clinic.

### Control ascertainment

Population-based controls (one per case) were matched on age (within 24 months), sex, and region of residence at the time (calendar date) of diagnosis. The controls were chosen from the most complete census of children for the study years, namely the family allowance files, awarded to all families with children in Canada. A list of 10 potential controls was randomly chosen from the lists according to the expected distribution of cases based on matching criteria.

### Study participants

We excluded children who were adopted, lived in foster families, whose family spoke neither French nor English, were not resident in Canada, or whose parents were both unavailable for interview. We identified 510 eligible cases and interviewed 491 parents (96.3%); 588 eligible controls were identified to obtain an interview from 493 (83.8%) parents. Among controls included in the study, 83% were first choices, 15% second, 1.8% third, and 0.2% were fourth choices. Reasons for non-participation among controls were a confidential telephone number (28%), refusal (38%), or the family could not be traced (34%). In the end, two strata without cases had to be rejected leaving 491 cases to be used in the analysis and 491 controls.

### Data collection

Soon after sending a letter introducing the general purpose to the study, trained interviewers contacted the parents of cases and controls to schedule an appointment for the interview, which was administered to the mother by telephone using a structured questionnaire. Questionnaires were reviewed as they came in and feedback was given regularly to interviewers.

For 48% of parents, the interview took place within 5 years of the date of the case's diagnosis (48.8% for cases and 47.7% for controls) and 84% and 86% of parents of cases and controls, respectively, were interviewed within 10 years of the date of the case's diagnosis.

The questionnaire included information on different potential markers of exposure to infection during pregnancy and infancy, namely: birth order and number of children in the family (and timing such as during pregnancy, first year of life and at time of diagnosis), maternal use of antibiotics during pregnancy, a history of recurrent infections in the mother, presence of a dog or cat in the house between pregnancy of the index child and diagnosis; day-care nursery attendance (timing relative to cases's date of diagnosis and hours of attendance/week) and principal type of feeding (breast or bottle) and if breast-feeding, duration. We had also asked about maternal blood transfusion during pregnancy (3 cases and 1 control reported positively); maternal vaccination during pregnancy (10 cases and 8 controls reported positively), and infection of the baby at birth (2 cases and 1 control). Because of the small numbers involved, these variables were not used in the analysis. From the questionnaire, we determined the number of changes of residence between birth and diagnosis. In addition, we determined whether there was at least one change of address for each of the following periods: pregnancy, first year of life and year of diagnosis. From the quinquennial censuses between 1976 and 1996, yearly population influx for all census divisions was estimated (Statistics Canada). We first created two variables: the first describes influx from anywhere (another country, another province in Canada or census division within the province) while the second describes influx from another country or province. We then estimated for each child a time-weighted average for each variable over the different census divisions in which the child had lived between birth and diagnosis including the pregnancy period, the first year of life and the year before diagnosis. Categories were then created contrasting a life-long stay in areas with an average influx of 5% or over versus a stay in areas with an average influx of less than 5% for the first variable (the range was between 1 and 10%) and an influx of more than 1% versus 1% or less for the second variable (the range was between 0 and 2%). The percentage came from dividing the yearly influx by the population denominator in the census division.

### Analysis

The association between markers of exposure and risk of ALL was estimated using conditional logistic regression. Odds ratios (OR) and 95% confidence intervals (95% CI) were estimated for the

© 2000 Cancer Research Campaign effect of each exposure variable (defined above) and for this effect adjusting for mother's age and education. In order to assess the potential effect of age at diagnosis on the observed associations, the analysis was done separately for two groups of children; cases and their paired controls diagnosed before 4 years of age and those diagnosed at 4 years or more. This cut-off point was chosen a priori as it corresponds to the beginning of preschool years and a time when social contacts for children are likely to change.

### RESULTS

The case and control groups each included 216 girls and 275 boys. In 465 pairs, the case and the matched control had an age difference of less than 3 months; the difference was between 3 and less than 6 months in 10 pairs, between 6 and less than 12 months in 8 pairs and 12 months or more in 8 pairs. There were slight differences between cases and controls in the distribution of mother's age at birth of the child and mother's education (Table 1).

The distribution of children according to potential markers of exposure to infection and breast-feeding is shown in Table 2, with odds ratios for these variables. There were no major differences between the ORs accounting only for the matching variables and those adjusted in addition for maternal age and level of schooling. Certain markers were associated with a significantly increased risk of ALL, such as having older or younger siblings, higher birth order and mother's use of antiobiotics during pregnancy. On the other hand, significantly protective effects were observed for day-care attendance at or before 2 years of age and for both durations of breast-feeing. Among children who attended day-care nursery, the duration of attendance was on average 27.7 hours/week for cases and 30.8 hours/week for controls. The number of years of attendance before diagnosis was on average 1.7 years for cases and 1.6 years for controls. Finally, living in regions with a greater population influx from anywhere outside the province was associated with a small and non-significant increase in risk; risk did not increase with a greater number of residences occupied.

We also carried out all the analyses included in Table 2 using only the pairs matched to within 3 months of age. Results were not materially different.

**Table 2** Odds ratios and 95% confidence intervals for markers of infections and population mixing in a case-control study of childhood acute lymphoblastic leukaemia

| Characteristics | Number of | | Crude | | Adjusted | |
|---|---|---|---|---|---|---|
| | Cases | Controls | OR[a] | 95% CI | OR[b] | 95% CI |
| Family structure (at diagnosis)[c] | | | | | | |
| Older siblings (yes vs no) | 256 | 179 | 2.17 | 1.62–2.91 | 2.12 | 1.57–2.85 |
| Younger siblings (yes vs no) | 152 | 155 | 1.41 | 1.01–1.94 | 1.43 | 1.03–1.98 |
| Birth order | | | | | | |
| 1 | 181 | 250 | 1.00 | – | 1.00 | – |
| 2 | 169 | 139 | 1.69 | 1.25–2.29 | 1.66 | 1.22–2.25 |
| ≥3 | 141 | 102 | 1.91 | 1.38–2.65 | 1.83 | 1.30–2.57 |
| Mother's use of antibiotics during pregnancy | | | | | | |
| No | 407 | 436 | 1.00 | – | 1.00 | – |
| Yes | 70 | 48 | 1.46 | 1.00–2.14 | 1.50 | 1.02–2.21 |
| Having a cat or a dog (before diagnosis) | | | | | | |
| No | 261 | 255 | 1.00 | – | 1.00 | – |
| Yes | 230 | 236 | 0.86 | 0.56–1.33 | 0.94 | 0.72–1.22 |
| Recurrent maternal infections | | | | | | |
| No | 450 | 452 | 1.00 | – | 1.00 | – |
| Yes | 36 | 31 | 1.14 | 0.68–1.92 | 1.09 | 0.65–1.84 |
| Attendance at day-care nursery (before diagnosis) | | | | | | |
| No | 398 | 353 | 1.00 | – | 1.00 | – |
| Entry at ≤2 years old | 35 | 63 | 0.49 | 0.31–0.76 | 0.49 | 0.31–0.77 |
| Entry at >2 years old | 57 | 75 | 0.65 | 0.44–0.97 | 0.67 | 0.45–1.01 |
| Breast-feeding | | | | | | |
| No | 282 | 239 | 1.00 | – | 1.00 | |
| ≤ 3 months | 104 | 125 | 0.67 | 0.49–0.93 | 0.68 | 0.49–0.95 |
| >3 months | 105 | 127 | 0.66 | 0.47–0.92 | 0.67 | 0.47–0.94 |
| Number of residences occupied (before diagnosis) | | | | | | |
| 1 | 179 | 196 | 1.00 | – | 1.00 | – |
| 2 | 165 | 139 | 1.29 | 0.95–1.75 | 1.35 | 0.99–1.85 |
| ≥3 | 147 | 156 | 1.04 | 0.76–1.41 | 1.04 | 0.79–1.48 |
| Living in census division with an average proportion of population influx (from anywhere) | | | | | | |
| <5% | 290 | 279 | 1.00 | – | 1.00 | – |
| ≥5% | 183 | 196 | 0.89 | 0.66–1.20 | 0.87 | 0.66–1.16 |
| Living in census division with an average proportion of population influx (from out of Canada or the rest of Canada) | | | | | | |
| <1% | 339 | 363 | 1.00 | – | 1.00 | – |
| ≥1% | 134 | 112 | 1.28 | 0.94–1.76 | 1.28 | 0.93–1.76 |

[a]Matched odds ratio. [b]matched odds ratio adjusted for maternal age and level of schooling. [c]Variables defining the measure are included together in the model.

© 2000 Cancer Research Campaign

**Table 3** Odds ratios[a] and 95% confidence intervals for markers of infection according to age at diagnosis in a case-control study of childhood acute lymphoblastic leukaemia

| | Age at diagnosis | | | | | | | |
|---|---|---|---|---|---|---|---|---|
| | <4 years old | | | | ≥4 years old | | | |
| | Number of | | OR[a] | 95% CI | Number of | | OR[a] | 95% CI |
| | Cases | Controls | | | Cases | Controls | | |
| Family structure[b] | | | | | | | | |
| Older siblings (yes vs no) | 141 | 68 | 3.59 | 2.30–5.60 | 115 | 111 | 1.31 | 0.84–2.05 |
| Younger siblings (yes vs no) | 45 | 62 | 1.00 | 0.60–1.69 | 107 | 93 | 1.50 | 0.96–2 34 |
| Mother's use of antibiotic during pregnancy | | | | | | | | |
| No | 201 | 220 | 1.00 | – | 206 | 216 | 1.00 | – |
| Yes | 42 | 25 | 1.78 | 1.04–3.04 | 28 | 23 | 1.29 | 0.72–2.31 |
| Attendance at day-care nursery | | | | | | | | |
| Never | 216 | 191 | 1.00 | – | 183 | 162 | 1.00 | – |
| Entry at ≤2 years old | 21 | 38 | 0.49 | 0.28–0.86 | 14 | 25 | 0.46 | 0.20–1.05 |
| Entry at >2 years old | 12 | 20 | 0.41 | 0.17–0.98 | 45 | 55 | 0.75 | 0.47–1.20 |
| Breast-feeding | | | | | | | | |
| No | 137 | 112 | 1.00 | – | 144 | 127 | 1.00 | – |
| ≤3 months | 54 | 68 | 0.62 | 0.37–1.03 | 50 | 57 | 0.78 | 0.50–1.23 |
| >3 months | 58 | 69 | 0.63 | 0.39–1.03 | 48 | 58 | 0.68 | 0.41–1.14 |

[a]Adjusted for maternal age and level of schooling in addition to matching variables; [b]variables defining the measure are included together in the model.

**Table 4** Odds ratios[a] and 95% confidence intervals for markers of infection according to age at diagnosis and timing of exposure in a case-control study of childhood acute lymphoblastic leukaemia

| Variables | Diagnosed at <4 years old (OR and 95% CI) | Diagnosed at ≥4 years old (OR and 95% CI) |
|---|---|---|
| Siblings[b] | | |
| At least 1 sibling during pregnancy | 1.13 (0.46–2.75) | 0.95 (0.43–2.09) |
| At least 1 sibling ≥4 years old during first year of life | 0.72 (0.26–2.00) | 0.46 (0.22–0.97) |
| At least 1 sibling ≥4 years old at time of diagnosis | 4.54 (2.27–9.07) | 2.08 (1.26–3.42) |
| Attendance at day-care nursery[b] | | |
| During the first year of life | 0.39 (0.15–1.04) | 0.47 (0.16–1.41) |
| In the year previous to diagnosis | 1.33 (0.64–2.76) | 1.28 (0.68–2.41) |
| Breast-feeding (baseline is none) | | |
| 1–6 months | 0.61 (0.39–0.95) | 0.72 (0.48–1.09) |
| >6 months | 0.68 (0.36–1.28) | 0.83 (0.42–1.63) |

[a]Adjusted for maternal age and level of schooling in addition to matching variables. [b]variables defining the measure are included together in the model.

An analysis similar to that in Table 2 was carried out comparing children diagnosed before 4 years of age and those diagnosed at 4 years of age and later; only variables with substantially increased or decreased odds ratios are shown (Table 3). Both variables about any other siblings were included together in the model. In the younger age group, having older siblings only was associated with a substantial increase in ALL risk, whereas this increase was small and not significant in children diagnosed at 4 or more years of age. There was a 50% marginally significant increase in risk associated with having younger siblings in children diagnosed at age 4 or later. The odds ratio for mother's use of antibiotics during pregnancy was also higher among the younger subgroup. The protective effects observed for children who attended day-care nursery at 2 years of age or before were similar in the two age groups though it was statistically significant only in the younger one. Finally, breast-feeding was protective in the younger age group though only marginally significant.

Timing of exposure to markers of infection appeared to be important (Table 4). Breast-feeding was defined using only one category. A school age sibling during the first year of life was protective, significantly so for those diagnosed at 4 years of age or later, whereas a school age sibling at the time of diagnosis increased the risk in both groups but in particular among those diagnosed before age 4. Early day-care attendance seemed to confer protection, although only the results for early attendance in the group diagnosed before 4 years of age were marginally significant. There was a protective effect for both durations of breast-feeding relative to none but it was strongest and statistically significant only for a duration of 1 to 6 months in the group diagnosed before age 4 (OR = 0.61; 95% CI 0.39–0.95).

Finally, we also considered the timing of exposure for time-weighted averages of population influx variables and the changes in residence for the whole group of study children. For the influx from anywhere, adjusting for maternal age and level of schooling, the ORs for ≥ 5 % were 1.48 (95% CI: 0.69–3.18) during pregnancy; 0.92 (95% CI 0.41–2.08) in the first year of life, and 0.71 (95% CI 0.40–1.23) in the year before diagnosis. For population

 © 2000 Cancer Research Campaign influx from out of Canada or the rest of Canada, the ORs for ≥ 1% for the same periods of exposure were: 0.77 (95% CI 0.39–1.51); 1.23 (95% CI 0.41–2.08); and 1.11 (95% CI 0.62–2.01), respectively. Changing residence during these same periods was associated with the following ORs: 0.88 (95% CI 0.64–1.21); 1.17 (95% CI 0.87–1.56); and 1.29 (95% CI 0.91–1.84), respectively.

### DISCUSSION

While the relation between infection and childhood leukaemia is increasingly recognized, little is known about the underlying mechanisms involved. However, genetic susceptibility, timing of exposure, and factors such as breast-feeding that act as modulators of the immune system may be important (Alexander, 1993; Greaves, 1997). Our results suggest that exposure to infection and its timing are important factors in the risk of ALL: early contact with infections through day-care and older siblings during the first year of life seemed protective of ALL, as well as breast-feeding during the first 6 months of life. However, the presence of older siblings at the time of diagnosis was a strong risk factor.

We observed a protective effect of breast-feeding lasting 3 or 6 months when all the study group was considered together regardless of age at diagnosis. A recent study reported for ALL an OR of 0.80 (95% CI 0.69–0.93) for children ever breast-fed; this protective effect was somewhat stronger with duration of breast-feeding longer than six months (Shu et al, 1999), a result which we did not confirm. A protective effect of breast-feeding on lymphoma was reported in other studies (Davis et al, 1988; Shu et al, 1995), but the effect was not significant for leukaemia (Davis et al, 1988; Magnani et al, 1988; Shu et al, 1995). Some of the components of breast milk actively stimulate the immune system and promote its development, increasing the capacity of children to face immune challenge (Hasselbalch et al, 1996; Hanson, 1998; Villalpando and Hamosh, 1998). This supports the immunological model proposed by Greaves (1997) and the present results.

In a day-care nursery environment, children are without doubt in contact with important doses of a wide range of infectious agents. First, the input of new infectious agents from parents, staff and children is common. Second, the transmission of infection is facilitated by continuous direct contacts between children and sharing of toys and facilities. The protective effect of early day-care attendance, in particular for those diagnosed before 4 years of age, observed in the present study is consistent with the hypothesis that early exposure to infectious agents would stimulate the immune system and provide better protection against later infections and consequently childhood leukaemia (Greaves, 1997).

In this study, the risk of leukaemia was decreased for children who attended day-care early, while it increased for later-born children and for children with older siblings, in particular for children diagnosed before 4 years of age. A further analysis showed that in both age groups at diagnosis (but more strongly in the younger one), the increased risk associated with older siblings was limited to the time of diagnosis. Having an older sibling during the first year of life, however, was protective in both groups though statistically significant only in the older group. The latter finding is consistent with the effect of early day-care attendance and the general hypothesis that early contact with infection is protective of leukaemia later. The finding about older siblings at the time of diagnosis emphasizes that timing of exposure to infections is important; this result suggests that as a high degree of social contacts in parents seems to increase the risk of childhood leukaemia (Kinlen et al, 1991; Kinlen, 1997), a similar influence for siblings is possible.

There is evidence that infections can be transmitted by the mother to the fetus through the placental barrier; transmission of virus possibly involved in childhood leukaemia such as the herpes virus family, Epstein-Barr virus and parvovirus B-19 was reported (Kaplan, 1990). The role of infection during pregnancy in the development of childhood leukaemia has frequently been addressed (Greaves, 1997; Smith et al, 1997; Smith, 1997) even though few studies have specifically evaluated the association (Roman et al, 1997; McKinney et al, 1999). Infection during pregnancy is suspected to represent an important element of the aetiology of childhood leukaemia, particularly for cases diagnosed before 5 years of age (Smith, 1997; Smith et al, 1997), which is consistent with our results.

Dickinson and Parker (1999) have recently provided strong support for the relation between population mixing and childhood leukaemia. Our results do not clearly support this hypothesis though the magnitude of population mixing in Quebec was certainly not as important as in northwest England and large population movement to isolated regions were not observed during the study period. Although our study covered a wide area it is possible, even though improbable, that matching on age, sex and region of residence could have influenced the observed association towards null value. On the other hand, our indicator of population mixing (influx averaged over census divisions, i.e. large regions) may not be sensitive enough to detect changes occurring in small areas. Population mixing is an imprecise marker of exposure to infection, though it is relevant that no example is known in the study area of the extreme rural population mixing studied by Kinlen (1995). Such markers as day-care attendance, number of siblings, use of maternal antibiotics or history of recurrent infections in the parents may afford this study better markers of individual exposure to infection.

The response rates and the source of data for control selection reduce the probability of selection bias. However, despite the use of a structured questionnaire and quality control measures, the likelihood of differential misclassification cannot be excluded. Although the delay between diagnosis (or reference for controls) and interview was the same for cases and controls, for many study subjects it was long and thus could have affected the quality of reporting. Parental recall bias leading to differential misclassification was evaluated for certain variables such as prenatal X-rays and distance from home to power lines (Infante-Rivard and Jacques, 2000); the results suggest that both cases and controls under-reported exposure but in a similar way leading to a bias towards the null. However, specific variables in the present analysis have not been the object of a validation study and thus differential misclassification in reporting cannot be eliminated.

In conclusion, the present results support the view that infection may be involved in the aetiology of childhood leukaemia and that timing of exposure is important.

### ACKNOWLEDGEMENTS

We wish to thank the oncologists and haematologists Drs Jean-Marie Leclerc (Hôpital Sainte-Justine), Mark Bernstein (Montreal Childrens’ Hospital), Linda Côté-Brisson (Centre Hospitalier de l’Université Laval), Josée Brossard (Centre Hospitalier de l’Université de Sherbrooke), and Reynald Simard (Centre Hospitalier de Chicoutimi) for their collaboration, and the following members of the

© 2000 Cancer Research Campaign research team: Marcelle Petitclerc, Denyse Hamer, Annie Chartier. We are indebted to Dr Louise Parker for her comments on a previous version of the manuscript.

This project was supported by grants from the National Research and Development Program, Ottawa and the Hydro-Québec-Fonds de la Recherche en Santé du Québec program on environment and child health.

### REFERENCES

Alexander FE (1993) Viruses, clusters and clustering of childhood leukaemia: a new perspective? *Eur J Cancer* **29A**: 1424–1443

Alexander FE, Ricketts TJ, McKinney PA and Cartwright RA (1990) Community lifestyle characteristics and risk of acute lymphoblastic leukaemia in children. *Lancet* **336**: 1461–1465

Alexander FE, Chan LC, Lam TH, Yuen P, Leung NK, Ha SY, YuenHL, Li CK, Li CK, Lau YL and Greaves MF (1997) Clustering of childhood leukaemia in Hong Kong: association with the childhood peak and common acute lymphoblastic leukaemia and population mixing. *Br J Cancer* **75**: 457–463

Davis MK, Savitz DA and Graubard BI (1988) Infant feeding and childhood cancer. *Lancet* **2**: 365–368

Dickinson HO and Parker L (1999) Quantifying the effect of population mixing on childhood leukaemia risk: the Seascale cluster. *Br J Cancer* **81**: 144–151

Greaves MF (1997) Aetiology of acute leukaemia. *Lancet* **349**: 344–349

Groves FD, Gridley G, Wacholder S, Schu XO, Robison LL, Neglia JP and Linet MS (1999) Infant vaccinations and risk of childhood acute lymphoblastic leukaemia in the USA. *Br J Cancer* **81**: 175–178

Hanson LA (1998) Breastfeeding provides passive and likely long-lasting active immunity. *Ann Allergy Asthma Immunol* **81**: 523–537

Hasselbalch H, Jeppesen DL, Engelmann MDM, Michaelsen KF and Nielsen MB (1996) Decreased thymus size in formula-fed infants compared with breastfed infants. *Acta Paediatr* **85**: 1029–1032

Infante-Rivard C and Jacques L. Empirical study of parental recall bias. (2000) *Am J Epidemiol* **152**: 480–486

Kaplan C (1990) The placenta and viral infections. *Clin Obstet Gynecol* **33**: 232–241

Kinlen LJ (1995) Epidemiological evidence for an infective basis in childhood leukaemia. *Br J Cancer* **71**: 1–5

Kinlen LJ (1997) High-contact paternal occupations, infection and childhood leukaemia: five studies of unusual population-mixing of adults. *Br J Cancer* **76**: 1539–1545

Kinlen LJ, Hudson CM and Stiller CA (1991) Contacts between adults as evidence for an infective origin of childhood leukemia: an explanation for the excess near nuclear establishments in West Berkshire? *Br J Cancer* **64**: 549–554

Kinlen LJ, Dickson M and Stiller CA (1995) Childhood leukaemia and non-Hodgkin's lymphoma near rural construction sites, with a comparison with Sellafield nuclear site. *Br Med J* **310**: 763–768

MacMahon B (1992) Is acute lymphoblastic leukemia in children virus-related? *Am J Epidemiol* **136**: 916–924

Magnani C, Pastor G and Terracini B (1988) Infant feeding and childhood cancer. *Lancet* **2**: 1136

McKinney PA, Juszczak E, Findlay E, Smith K and Thomson CS (1999) Pre- and perinatal risk factors for childhood leukaemia and other malignancies: a Scottish case control study. *Br J Cancer* **80**: 1844–1851

Neglia JP, Linet MS, Shu XO, Severson RK, Potter JD, Mertens AC, Wen W, Kersey JH and Robison LL (2000) Patterns of infection and daycare utilization and risk of childhood acute lymphoblastic leukemia. *Br J Cancer* **82**: 234–240

Petridou E, Kassimos D, Kalmanti M, Kosmidis H, Haidas S, Flytzani V, Tong D and Trichopoulos D (1993) Age of exposure to infections and risk of childhood leukaemia. *Br Med J* **307**: 774

Petridou E, Revinthi K, Alexander FE, Haidas S, Koliouskas D, Kosmidis H, Piperopolou F, Tzortzatou F and Trichopoulos D (1996) Space-time clustering of childhood leukaemia in Greece: evidence supporting a viral aetiology. *Br J Cancer* **73**: 1278–1283

Roman E, Ansell P and Bull D (1997) Leukaemia and non-Hodgkin's lymphoma in children and young adults: are prenatal and neonatal factors important determinants of disease? *Br J Cancer* **76**: 406–415

Ross JA, Davies SM, Potter JD and Robison LL (1994) Epidemiology of childhood leukemia, with a focus on infant. *Epidemiol Rev* **16**: 243–272

Shu XO, Linet MS, Steinbuch M, Wen WQ, Buckley JP, Neglia JP, Potter JD, Reaman GH and Robison LL (1999) Breast-feeding and risk of childhood acute leukemia. *J Natl Cancer Inst* **91**: 1765–1772

Shu X, Clemmens J, Zheng W, Ying DM, Ji BT and Jin F (1995) Infant breastfeeding and the risk of childhood lymphoma and leukaemia. *Int J Epidemiol* **24**: 27–32

Smith M (1997) Considerations on a possible viral etiology for B-precursor acute lymphoblastic leukemia of childhood. *J Immunother* **20**: 89–100

Smith MA, Chen T and Simon R (1997) Age-specific incidence of acute lymphoblastic leukemia in U.S. children: In utero initiation model. *J Natl Cancer Inst* **89**: 1542–1544

Smith MA, Simon R, Strickler HD, McQuillan G, Gloeckler Ries LA and Linet MS (1998) Evidence that childhood acute lymphoblastic leukemia is associated with an infectious agent linked to hygiene conditions. *Cancer Causes Control* **9**: 285–298

Statistics Canada (year ?) Canadian Socio-Economic Information Management System: matrices 6486–6493; 6191–6198

Stiller CA and Boyle PJ (1996) Effect of population mixing and socioeconomic status in England and Wales, 1979–85, on lymphoblastic leukaemia in children. *Br Med J* **313**: 1297–1300

Villalpando S and Hamosh M (1998) Early and late effects of breast-feeding: does breast-feeding really matter? *Biol Neonate* **74**: 177–191

Westergaard T, Andersen PK, Pedersen JB, Olsen JH, Frisch M, Sorensen HT, Wohlfahrt J and Melbye M (1997) Birth characteristics, sibling patterns, and acute leukemia risk in childhood: a population-based cohort study. *J Natl Cancer Inst* **89**: 939–947

 © 2000 Cancer Research Campaign
